# Identification and characterization of repetitive extragenic palindromes (REP)-associated tyrosine transposases: implications for REP evolution and dynamics in bacterial genomes

**DOI:** 10.1186/1471-2164-11-44

**Published:** 2010-01-19

**Authors:** Jaroslav Nunvar, Tereza Huckova, Irena Licha

**Affiliations:** 1 Department of Genetics and Microbiology, Faculty of Science, Charles University, Vinicna 5, 128 44 Prague 2, Czech Republic

## Abstract

**Background:**

Bacterial repetitive extragenic palindromes (REPs) compose a distinct group of genomic repeats. They usually occur in high abundance (>100 copies/genome) and are often arranged in composite repetitive structures - bacterial interspersed mosaic elements (BIMEs). In BIMEs, regularly spaced REPs are present in alternating orientations. BIMEs and REPs have been shown to serve as binding sites for several proteins and suggested to play role in chromosome organization and transcription termination. Their origins are, at present, unknown.

**Results:**

In this report, we describe a novel class of putative transposases related to IS*200*/IS*605 *transposase family and we demonstrate that they are obligately associated with bacterial REPs. Open reading frames coding for these REP-associated tyrosine transposases (RAYTs) are always flanked by two REPs in inverted orientation and thus constitute a unit reminiscent of typical transposable elements. Besides conserved residues involved in catalysis of DNA cleavage, RAYTs carry characteristic structural motifs that are absent in typical IS*200*/IS*605 *transposases. DNA sequences flanking *rayt *genes are in one third of examined cases arranged in modular BIMEs. RAYTs and their flanking REPs apparently coevolve with each other. The *rayt *genes themselves are subject to rapid evolution, substantially exceeding the substitution rate of neighboring genes. Strong correlation was found between the presence of a particular *rayt *in a genome and the abundance of its cognate REPs.

**Conclusions:**

In light of our findings, we propose that RAYTs are responsible for establishment of REPs and BIMEs in bacterial genomes, as well as for their exceptional dynamics and species-specifity. Conversely, we suggest that BIMEs are in fact a special type of nonautonomous transposable elements, mobilizable by RAYTs.

## Background

Transposable elements (TEs), or transposons, are a large group of mobile genetic elements with ability to actively transfer themselves into new locations in their host´s DNA. This process, called transposition, is catalyzed by transposases, coded for by TEs themselves. Insertion sequences (ISs) present the simplest examples of TEs.

The IS*200*/IS*605 *family of transposable elements was first described in genus *Salmonella *[1] and further in many other bacterial and archaeal genomes [2]. Contrary to the majority of TEs that transpose using transposases whose active site is composed of a triad of acidic residues (DDE transposases), known members of the IS*200*/IS*605 *family lack terminal inverted repeats and do not generate larger target site duplications upon transposition [3]. Crystal structures of two IS*200*/IS*605 *transposases have been solved (PDB IDs: 2a6o and 2f4f) [4,5]. Their fold is remarkably similar to proteins involved in rolling circle (RC) replication - conjugative plasmid relaxases and viral Rep proteins [4,5]. This similarity is further supported by shared mechanism of DNA cleavage: transesterification reaction takes place between DNA strand and conserved tyrosine residue, resulting in covalent protein-DNA intermediate. A histidine-hydrophobic-histidine motif and a divalent metal (magnesium) cation are another mandatory components of properly assembled active site, aiding the nucleophilic attack of catalytic tyrosine [6,7]. Next trait common for both IS*200*/IS*605 *transposases and RC enzymes is that the cleavage of DNA depends on the recognition of stem-loop structures, present at either the origin of RC replication or IS termini [6,7]. IS*200*/IS*605 *transposases are the smallest transposases known, with average length below 150 amino acids. To encompass size limitation, they work as a homodimer with two hybrid active sites, each composed of tyrosine from first unit and the histidine-hydrophobic-histidine motif from second unit [4,5].

As determination of eukaryotic genomic sequences progressed in the last two decades, it has become obvious that their genetic information is littered with highly repetitive, "junk" DNA. More detailed analyses of these repetitive elements revealed that many of them are actually special cases of TEs. They generally retain conserved terminal sequences (for example inverted repeats) of their corresponding full-length transposons, which are important for transposition initiation, but lack completely or partially the transposase gene. Therefore, transposase encoded by "parental" full-length transposons needs to be supplied *in trans*. These repetitive elements are thus called nonautonomous TEs. Three groups of nonautonomous TEs account for substantial fractions of eukaryotic genomes. The first group is represented by short interspersed nuclear elements (Alu-like), derived from non-LTR-retrotransposons [8]. Helitrons, the second type of nonautonomous TEs, are thought to be mobilized by Y-2 type transposases, that are homologous to RC replication relaxases [9]. The last type, miniature inverted repeat transposable elements (MITEs), is present in both eukaryotes and prokaryotes. Most studied MITEs are related to two homologous insertion sequence families, IS*630 *(prokaryotic) and Tc-Mariner (eukaryotic) [10], both employing DDE catalytic mechanism. IS*630*-derived MITEs in prokaryotic genomes include Correia elements in *Neisseria *species [11] and RUP elements in *Streptococcus pneumoniae *[12]. Besides these, MITEs related to other IS families have been identified in prokaryotes [2].

Repetitive extragenic palindromic sequences (REPs) were originally identified in enteric bacteria [13] and later in several other bacterial taxa [14-16] as a class of abundant repeats with characteristic architecture. REP elements contain imperfect palindrome in their sequence. The majority of REPs are arranged in repeats of higher order, bacterial interspersed mosaic elements (BIMEs) [17]. In BIME-1, two oppositely orientated REPs are located close to each other. The inter-REP sequence interacts with integration host factor (IHF) [18]. BIME-2 and atypical BIMEs are composed of several tandemly repeated BIME-1-like units [19] and have been shown to strongly bind DNA gyrase [20]. REPs themselves interact with DNA polymerase I [21] and facilitate Rho-dependent transcription termination [22].

Our present results describe an intimate relationship between REP and BIME elements and one apparently monophyletic group of IS*200*/IS*605 *transposases. Because of striking similarities to known nonautonomous TEs, we propose that BIMEs are in fact nonautonomous TEs and that IS*200*/IS*605 *transposases are responsible for their mobilization.

## Results

### Case study - genus *Stenotrophomonas*

**Figure 1:**
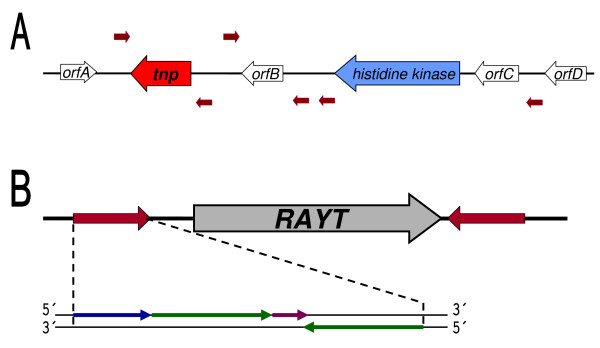
***Stenotrophomonas *RAYTs**. (A) Schematic representation of a segment of *S. maltophilia R551-3 *genome containing putative IS*200*/IS*605 *family transposase gene (orange arrow), histidine kinase/response regulator/sodium-symporter family gene (blue arrow) and several short palindromic repeats (red arrows). (B) General structure of *Stenotrophomonas rayt *genes flanked by REPs. The whole REPs and their orientation are denoted with red arrows. Details in REP structure (bottom) are marked with arrows: blue - GT(A/G)G head, green - palindrome-forming sequence, pink - noncomplementary middle part of palindrome.

We have studied mechanisms of high-level tetracycline resistance in bacteria from agricultural soil treated with manure from tetracycline-fed animals. Among tetracycline-resistant isolates, identified as *Stenotrophomonas maltophilia*, *Variovorax paradoxus *and *Chryseobacterium balustinum*, horizontal gene transfer from *S. maltophilia *to other two species was detected. The transferred nucleotide sequence was 90% identical to a histidine kinase/response regulator/sodium-symporter family gene, present in both sequenced *S. maltophilia *strains. We investigated the region surrounding this gene in sequenced stenotrophomonads for the presence of genes known to be involved in horizontal transfer of genetic information. A putative transposase of the IS*200*/IS*605 *family was found one gene away from histidine kinase in *S. maltophilia R551-3*. Analysis of sequences flanking the transposase gene revealed inverted repeats containing an imperfect palindrome. More sequences identical to these inverted repeats were observed scattered in several instances between neighboring genes (Figure 1A).

We performed a BLAST search that revealed five apparent homologs of this transposase in genomes of sequenced stenotrophomonads. Their genes were all found to be delimited by inverted repeats of the same architecture (Figure 1B). The 5-GT(A/G)G "head" is immediately followed by perfectly complementary, GC-rich palindrome, interrupted by 2-4 bases in its middle (Table 1, bottom). Due to the presence of multiple copies of these repeated sequences in the proximity of the transposase gene (see above), we scanned whole *Stenotrophomonas *genomes for additional copies of repeats flanking each particular transposase homolog. The number of hits ranged from 37 up to 427 perfect copies of given repeat per genome (Table 1, bottom). Because of their palindromic nature and abundance, features they share with published REP sequences, they will be called REPs and their cognate transposases will be called REP-associated tyrosine transposases (RAYTs).

**Table 1:** Summary information on identified RAYTs and REPs

Host strain	RAYT symbol	RAYT accession	REP sequence^A)^	**Nr. of REP copies **^**B)**^	*rayt*-BIME association
*Citrobacter koseri ATCC BAA-895*	Ckos	YP_001455335	**GTAG**GCCcGgTAAGCGaaGCGCCaCCgGGC **GTAG**GCCgGaTAAgGCGcttGCGCCgCCatccGGC	9/46/77 16/23/65	N

*Enterobacter sakazakii ATCC BAA-894*	Esak	YP_001437784	**GTAG**GGcGGGTAAGCGgAGCGCACCCgCC **GTAG**GGtGGGTAAGCGcAGCGCACCCaCC	3/89/159 39/117/180	N

*Escherichia coli str. K-12 substr. MG1655*	Ecol	NP_414763	**GTAG**GacgGATAAGgCGttCACGcCGCATCcGGCA **GTAG**GcatGATAAGaCGcgcCAgCGtCGCATCaGGCA	4/52/126 4/19/46	**Y **

*Salmonella enterica subsp. enterica serovar Typhi str. CT18*	Sent	NP_458983	T**GTAG**GCCGGATAAGgcgtagcCGCCATCCGGC T**GTAG**GCCGGATAAGcaacgCGCCATCCGGC	1/10/16 1/2/6	N

*Klebsiella pneumoniae 342*	Kpne	YP_002239241	**GTAG**GCCcggcAAGCGcAGCGCCgccgGGC **GTAG**GCCggatAAgGCGaAGcCGCCatccGGC	8/12/20 2/11/13	**Y **

*Haemophilus parasuis SH0165*	Hpar	YP_002476161	**GTAG**GGTGGGTCTTGACCCACC	20/22/42	N

*Haemophilus influenzae Rd KW20*	Hinf	NP_438385	**GTAG**GGTGGGCTTcAGCCCACC **GTAG**GGTGGGCTTtAGCCCACC	6/14/21 5/16/21	**Y **

*Coxiella burnetii Dugway 5J108-111*	Cbur	YP_001425023	**GTAG**GTTGGGCTGAGCTTGCGAAGCCCAAC	29/38/40	N

*Thioalkalivibrio sp. HL-EbGR7*	T_sp	YP_002514838	**GTAG**GTCGGCCTTCAGGCCGAC	38/53/118	N

*Pseudomonas mendocina ymp*	Pmen	YP_001186231	**GTAG**CCCGGATGCAATCCGGG	75/136/162	N

*Pseudomonas putida KT2440*	Pput1	NP_747277	T**GTGG**GAGCGGGCgTGCCCGCGAA T**GTGG**GAGCGGGCaTGCCCGCGAA	62/193/286 35/172/285	N

*Pseudomonas putida GB-1*	Pput2	YP_001671454	T**GTGG**GAGCGGGTTtACCCGCGAA T**GTGG**GAGCGGGTTcACCCGCGAA	62/95/130 14/80/104	N

*Pseudomonas putida KT2440*	Pput3	NP_742731	T**GTGG**GAGCGGCCTTGcGTCGCGA T**GTGG**GAGCGGCCTTGtGTCGCGA	21/53/69 27/62/72	N

*Pseudomonas putida W619*	Pput4	YP_001751446	T**GTAG**GAGCGGCCTTGcGTCGCGAA T**GTAG**GAGCGGCCTTGtGTCGCGAA	24/112/189 77/174/205	**Y**

*Pseudomonas entomophila L48*	Pent1	YP_608776	T**GTAG**GAGCGGATTCATCCGCGAT	116/171/443	N

*Pseudomonas entomophila L48*	Pent2	YP_610581	**GTAG**GAGCCAGCTTGCTGGCGAA	89/101/564	N

*Pseudomonas fluorescens SBW25*	Pflu1	YP_002873491	**GTGG**GAGGGGGCTTGCCCCCGAT	387/557/607	N

*Pseudomonas fluorescens SBW25*	Pflu2	YP_002871781	**GTGG**CGAGGGAGCTTGCTCCCGCT	104/192/232	**Y**

*Pseudomonas fluorescens SBW25*	Pflu3	YP_002873800	T**GTgG**TGAGCGGGCTTGCCCCGCGCT T**GTaG**TGAGCGGGCTTGCCCCGCGCT	83/217/263 119/229/257	**Y**

*Xanthomonas axonopodis pv. citri str. 306*	Xaxo	NP_641493	**GTAG**GAGCGCACCtGGGCGCGAC **GTAG**GAGCGCACCcGGGCGCGAC	9/49/85 23/58/88	**Y **

*Xanthomonas campestris pv. campestris str. ATCC 33913*	Xcam	NP_636415	**GTAG**GAGCGCGCTCGCGCGCGA	48/177/223	**Y **

*Stenotrophomonas maltophilia R551-3*	Smal1	YP_002030358	T**GTAG**AGCCGAGCCCATGCTCGGCT	49/90/113	N

*Stenotrophomonas maltophilia R551-3*	Smal2	YP_002029847	G**GTAG**CGCCGGGCCATGCCCGGCG	259/329/355	N

*Stenotrophomonas maltophilia K279a*	Smal3	YP_001970973	G**GTGG**GTGCCGACCGTTGGTCGGCAC	52/75/99	N

*Stenotrophomonas maltophilia K279a*	Smal4	YP_001972572	G**GTAG**TGCCGGCCGCTGGCCGGCA	427/556/644	**Y **

*Stenotrophomonas sp. SKA14 *	S_sp1	YP_002706198	A**GTAG**ATCCACGCCATGCGTGGAT	69/147/182	N

*Stenotrophomonas sp. SKA14 *	S_sp2	YP_002708831	G**GTGG**GTGCCAACCTTGGTTGGCAC	37/84/131	N

(A) REP sequences, as found flanking the *rayt *gene, in 5´→3´ direction. When upstream and downstream REPs differ, both are denoted (differing bases in lower case). Palindromic parts are underlined. Conserved head sequences are written in bold. (B) Number of REP sequences in host strain's genome, in following order: completely identical copies/copies with 1 mismatch/copies with 2 mismatches.

**Figure 2:**
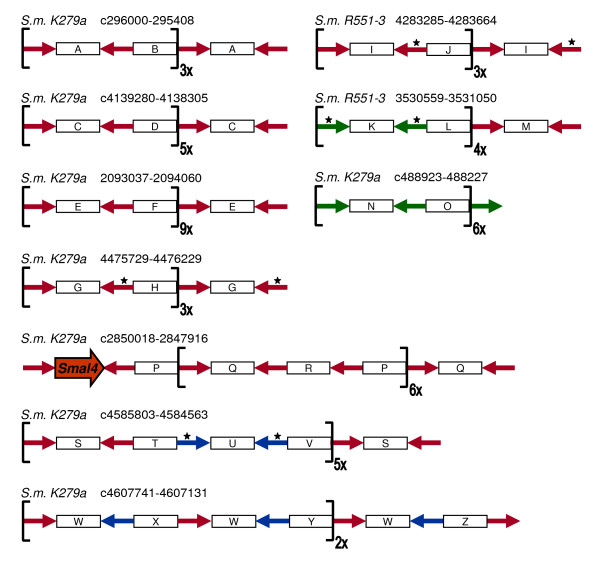
**Schematic representation of *S. maltophilia R551-3 *and *S. maltophilia K279a *BIMEs**. Host strain is indicated, followed by BIME coordinates. Each unique inter-REP sequence is assigned a different letter. Basic modules are bracketed, their numbers are denoted. REPs and their orientation are marked with arrows: red - Smal4 REP, green - Smal3 REP, blue - S_sp2 REP. Asterisks indicate modified REPs. Large orange arrow denotes gene coding for Smal4 RAYT.

We noticed that some of the REPs identified were arranged in clusters. Ten clusters composed of REPs were then analysed in detail (Figure 2). The core (basic module) of each of these compound structures consists at least of two inverted REPs, separated by two intervening segments. Several of these basic modules are connected to each other in a head-to-tail fashion. The inter-REP segments do not show any homology with each other and vary substantially in length, suggesting that these clusters arose repeatedly and independently. Because of their exceptional structural similarities with published BIMEs, they will be called BIMEs.

*Stenotrophomonas *BIMEs show several interesting aspects. Some of them are hybrid and contain REPs from two different RAYTs*. *Moreover, slightly modified REPs occur in BIMEs, differing only in a few nucleotide positions. Still, in all cases, the palindromic features of REPs are preserved, suggesting selection for complementary mutations. Intriguingly, one *rayt *gene (Smal4) is directly associated with a BIME, its downstream REP being one of the BIME-constituting REPs.

**Figure 3:**
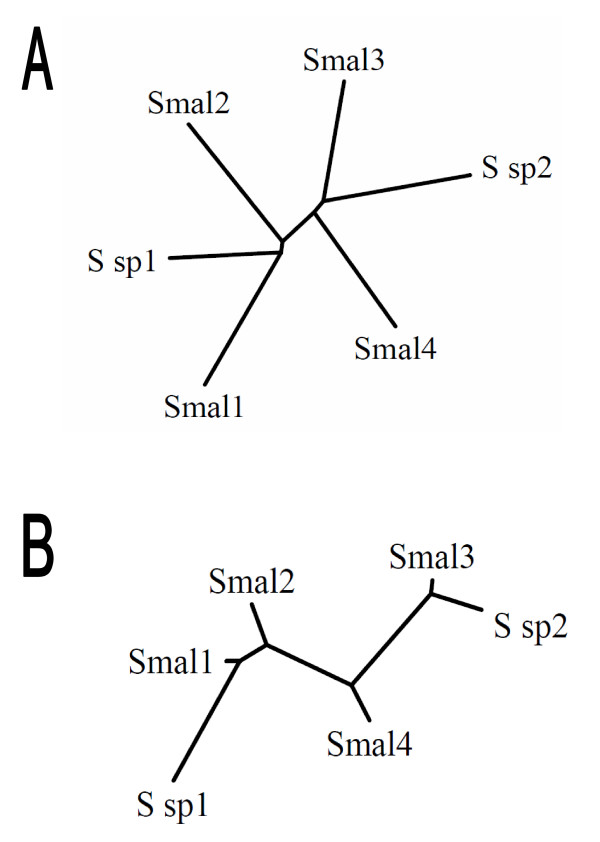
**Coevolution of RAYTs and REPs**. Unrooted phylograms, constructed from (A) *Stenotrophomonas *RAYT amino acid sequences and (B) *Stenotrophomonas *REP nucleotide sequences.

Since all six *rayt *genes are flanked by two inverted REPs, this type of organization is likely to be subject to evolutionary preservation. To estimate evolutionary relationship between these elements, phylogenetic trees were constructed from RAYT amino acid sequences and REP nucleotide sequences, respectively. Both phylograms display the same topology (Figure 3), suggesting that RAYTs coevolve with their cognate REPs and that their typical organization is ancestral.

### RAYTs in other bacteria

We wondered if similar RAYTs, REPs and BIMEs also occur together in other bacterial taxa. Using Smal1 RAYT sequence as query, exhaustive BLAST search was performed to identify RAYT homologs in other prokaryotic organisms. Retrieved homologs, all of which contained the "Pfam01797: Transposase_17" domain (peculiar to IS*200*/IS*605 *transposases), were tested for the presence of palindrome-containing inverted repeats flanking their genes. Subsequently, the number of these putative REPs in host genomes was determined. Only RAYTs associated with abundant REPs were further analysed. Detected RAYTs are listed in Table 1. RAYT homologs suiting our criteria were only found in gammaproteobacteria.

All detected REPs consist of GT(A/G)G head and GC-rich imperfect palindrome with potential to form stem-loop structures in single-stranded state (Table 1). Importantly, in all cases when REP sequences were determined in bacterial species taken into our analysis prior to this work, REPs identified by our approach are in agreement with these sequences. This concerns *Escherichia coli *[19]*, Salmonella sp. *[23]*, Pseudomonas putida *Pput2 [16] and *Stenotrophomonas maltophilia *Smal4 [24] REPs. For example, *E. coli *RAYT-coding gene (*yafM*) is delimited by two different REPs (Table 1). These are in fact Y and Z2 palindromic units, constituents of modular BIMEs (BIME-2 and atypical BIMEs) [25]. *E. coli rayt *itself is flanked by BIME-2 on both sides. Similar direct association with BIME was observed in total for one third of detected RAYTs (Table 1) in various species.

Further, we examined distribution of identified REPs in host genomes. Analysis revealed that most REPs are arranged in clusters (Additional file 1). In some cases (pseudomonads, *Thioalkalivibrio sp.*), the most predominant type of clusters is a doublet of REPs in inverted orientation. These REP doublets, together with embedded inter-REP sequences, might themselves represent compound repeated elements, analogous to *E. coli *BIME-1. This is supported by structure of recently described *Pseudomonas fluorescens *repeats [26]. The R0 family consists of 612 repeats (89 bp in length) that have two inverted elements at their termini, identical with Pflu1 REPs.

In contrast to the doublet arrangement, *Xanthomonas campestris *REPs are in great majority found in large clusters, consisting of regularly spaced REPs in alternating orientations (Additional file 1), typical features of BIMEs. In remaining cases, solitary REPs are found along with doublets and BIMEs.

Preliminary analysis confirmed that the great majority of all identified REPs are extragenic (data not shown) and thus further fulfill the definition of REP elements.

### Evolution of RAYTs and REPs

**Figure 4:**
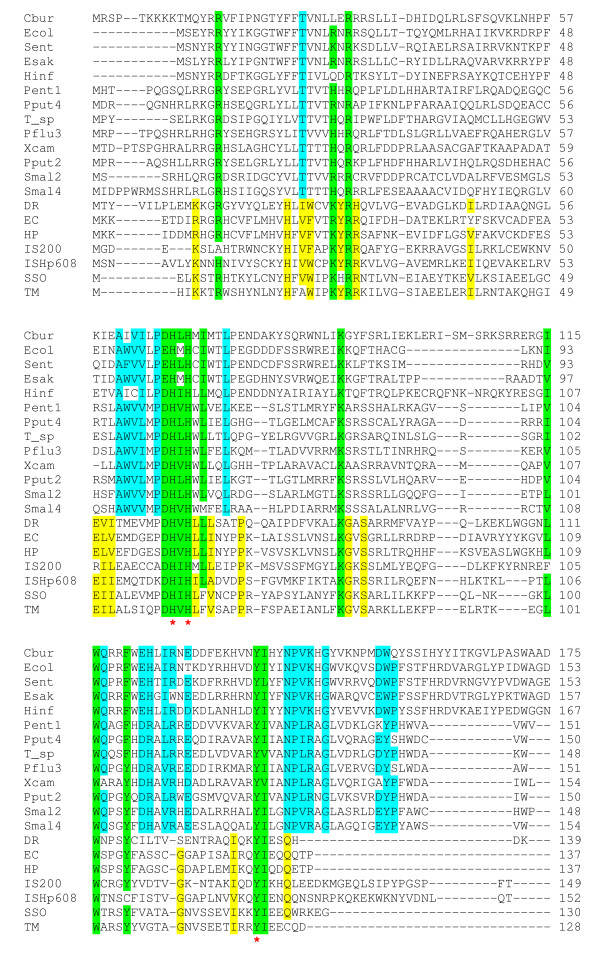
**Multiple sequence alignment of selected RAYTs and reference set of IS*200*/IS*605 *family transposases**. Conserved residues are highlighted: blue - conserved in RAYTs, yellow - conserved in reference IS*200*/IS*605 *transposases, green - conserved in both groups. Substitutions for residues with similar chemical properties are permitted: acidic - D, E, basic - H, K, R, aromatic - F, Y, W, branched-chain hydrophobic- I, L, V. Conserved residues that constitute catalytic center are denoted with red asterisk. Reference set of IS*200*/IS*605 *transposases, along with their symbols, was taken from [5].

Since REPs share several common structural features, they are likely to represent a group of related elements. We wondered if the same is true for RAYTs. Because RAYTs were detected due to similarity of their protein sequences (see above), they are thought to be structurally related. To specify this relationship, an alignment of selected RAYTs together with reference set of "typical" IS*200*/IS*605 *transposases was constructed (Figure 4). The alignment reveals that all catalytically confirmed residues - histidine-hydrophobic-histidine motif and nucleophilic tyrosine - are conserved in both groups. It is thus reasonable to conclude that RAYTs are capable of cleaving DNA with formation of DNA-RAYT covalent intermediate. On the contrary, several motifs and conserved residues are peculiar only to RAYTs. This is in particular true for 100% conserved threonine near N-terminus and the NP(L/V)(R/K)xG motif that is located close to C-terminus adjacently to nucleophilic tyrosine.

The presence of these unique structural features could signify that RAYTs are monophyletic group of proteins. The question therefore arises as to whether the entire RAYT clade has been evolving with their corresponding REPs, as seen in *Stenotrophomonas *(Figure 3). Due to rather high divergence of REPs, it is not possible to construct their accurate phylogram. However, REPs show group-specific features that correlate well with phylogenetic grouping of their cognate RAYTs. For example, enterobacterial RAYTs are clearly monophyletic (Additional file 2) and accordingly, their REPs are rather long, substantially dimorphic and their palindrome is interrupted twice (Table 1). Furthermore, uniquely for REPs of monophyletic *Pseudomonas *and *Xanthomonas *RAYTs (Additional file 2), 5´-GA-3´ dinucleotide is inserted between their GT(A/G)G head and palindrome-forming part (Table 1). Together, these observations support long-term coevolution of RAYTs and and their cognate REPs.

**Figure 5:**
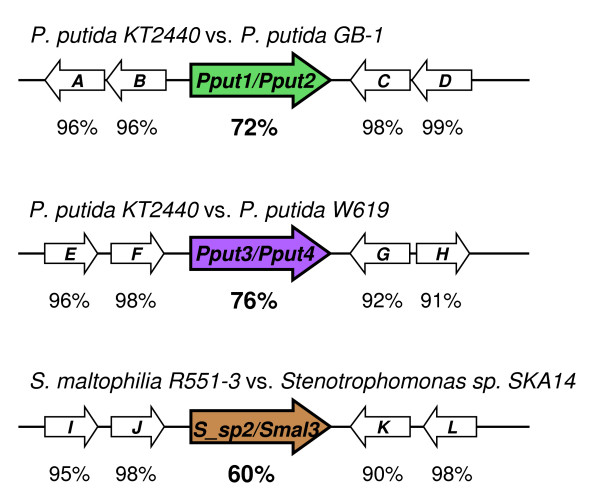
**Sequence identity between orthologous RAYTs and proteins coded for by neighboring orthologous genes**.

Next, we examined chromosomal localization of *rayt *genes. Among RAYTs listed in Table 1, three couples of orthologous *rayt *genes (Pput1 and Pput2, Pput3 and Pput4, Smal3 and S_sp2), located in the same genomic context in different host species or strains, were identified (Figure 5). These orthologs have, due to the shared synteny, unambiguously evolved from a common ancestor and allow us to trace back changes they have gone through following divergence event. Although orthologous *rayt *genes do not change their genomic position, their flanking REPs differ in up to three point mutations (Table 1) and still retain palindromicity and inverted repeat arrangement. Evidently, strong selective pressure works for preservation of these REP traits, underlining their functional importance. It is extremely improbable that repeated changes in REP sequences flanking these orthologs result merely from random fixation of successive random mutations.

Comparison of sequence identity between orthologous *rayt *genes revealed an interesting phenomenon. In all three cases, the degree of identity of the RAYT amino acid sequences was significantly less than that of the flanking genes (Figure 5), suggesting that RAYTs evolve more faster than protein products of common genes. Possible explanation for this accelerated evolution is included in the Discussion section.

### Relationship of RAYTs, REPs and BIMEs

We have shown so far that RAYTs and REPs are evolutionarily and physically connected. Since REPs are known to be species (or strain)-specific and the same applies to RAYTs (Table 1), it is possible that the presence of a particular RAYT itself in one bacterium might be responsible for proliferation of corresponding REPs.

Where genome sequences suitable for comparison were available, strains differing by the presence or absence of a particular *rayt *gene were tested for prevalence of REPs in their genomes. In most cases, a strong correlation between *rayt *presence and total number of its cognate REPs was found (Table 2), *rayt*-bearing strains containing on average ten times more REPs in their genomes than strains devoid of *rayt *genes. These results indeed suggest that presence of a given RAYT is the direct cause of REP sequences proliferation over host chromosome.

**Table 2:** Correlation between REP numbers and presence or absence of their cognate RAYTs in different bacterial strains

	REP symbol														
	
Host strain	**Pput1**	**Pput2**	**Pput3**	**Pput4**	**Pflu1**	**Pflu2**	**Pflu3**	**Xaxo**	**Xcam**	**Smal1**	**Smal2**	**Smal3**	**Smal4**	**S_sp1**	**S_sp2**
*Pseudomonas putida KT2440*	**62** **35**	4 3	**21** **27**	0 0	0	0	0 0	0 0	0	0	0	0	0	0	0

*Pseudomonas putida F1*	**70** **30**	4 4	**56** **78**	0 0	0	0	0 0	0 0	0	0	0	0	0	0	0

*Pseudomonas putida GB-1*	16 4	**62** **14**	4 23	6 63	0	0	0 0	0 0	0	0	0	0	0	0	0

*Pseudomonas putida W619*	0 0	1 1	0 0	**24** **77**	0	0	0 0	0 0	0	0	0	0	0	0	0

*Pseudomonas fluorescens SBW25*	0 0	0 0	0 0	0 0	**387**	**104**	**83** **119**	0 0	0	0	0	0	0	0	0

*Pseudomonas fluorescens Pf0-1*	0 0	0 0	0 0	0 0	0	0	0 0	0 0	0	0	0	0	0	0	0

*Pseudomonas fluorescens Pf-5*	0 0	0 0	0 0	0 0	0	1	0 0	0 0	0	0	0	0	0	0	0

*Xanthomonas axonopodis pv. citri str. 306*	0 0	0 0	0 0	0 0	0	0	0 0	**9** **23**	0	0	0	0	0	0	0

*Xanthomonas. campestris pv. vesicatoria str. 85-10*	0 0	0 0	0 0	0 0	0	0	0 0	**7** **11**	1	0	0	0	0	0	0

*Xanthomonas campestris pv. campestris str. 8004*	0 0	0 0	0 0	0 0	0	0	0 0	0 3	**43**	0	0	0	0	0	0

*Xanthomonas. campestris pv. campestris str. ATCC 33913*	0 0	0 0	0 0	0 0	0	0	0 0	1 3	**48**	0	0	0	0	0	0

*Xanthomonas campestris pv. campestris str. B100*	0 0	0 0	0 0	0 0	0	0	0 0	2 4	**49**	0	0	0	0	0	0

*Stenotrophomonas maltophilia R551-3*	0 0	0 0	0 0	0 0	0	0	0 0	0 0	0	**49**	**259**	**39**	266	25	4

*Stenotrophomonas maltophilia K279a*	0 0	0 0	0 0	0 0	0	0	0 0	0 0	0	7	13	**52**	**427**	47	16

*Stenotrophomonas sp. SKA14*	0 0	0 0	0 0	0 0	0	0	0 0	0 0	0	3	7	7	**323**	**69**	**37**

The values represent the numbers of exact copies of REP sequences, flanking identified *rayt *genes (as denoted in Table 1), in bacterial genomes. For dimorphic REPs, the upper value corresponds to the number of upper REP sequences from Table 1 and *vice versa*. In cases where the cognate *rayt *gene or its close homolog (flanked by the same REPs) is actually present in the given genome, the numbers are written in bold and underlined.

In search of support for this hypothesis, we found that in three marine gammaproteobacteria and one betaproteobacterium (all possessing clear RAYT homologs), the distribution of inverted palindromic repeats flanking their *rayt *genes is not genome-wide (as in other REP cases). Instead, REPs are accumulated proximally to particular *rayt *gene (Additional file 3). The REP-containing regions span at most two hundreds of kilobases. In the case of the marine gammaproteobacteria, the physical association between *rayt *genes and REPs is very pronounced. *Thauera sp. *(a betaproteobacterium) is of special interest because it has obviously acquired its RAYT by horizontal transfer from gammaproteobacteria. This RAYT displays highest sequence similarity to *Pseudomonas *RAYTs (56% identical residues), has no counterpart in other betaproteobacteria and its REP sequences are also *Pseudomonas*-like (Table 1, Additional file 3). High numbers of REPs are present in the *Thauera *genome. More than a third are located proximally to *rayt *gene. This suggests that, following acquisition of the *rayt *gene, new REP copies have been preferentially produced in its vicinity.

Physical association with *rayt *genes was already shown for BIMEs (Table 1). Upon closer examination, we detected four cases where 3´ end of *rayt *gene, together with sequence between *rayt *stop codon and downstream REP, is integrated into BIME, becoming a part of BIME´s inter-REP segment (Additional file 4). This unexpected observation proves that the mechanism responsible for establishment of BIMEs is also directed to *rayt *genes.

## Discussion

We have characterized a novel class of transposases, closely related to IS*200*/IS*605 *family. What makes these transposases (RAYTs) unique is the obligate delimitation of their genes by two inverted palindromic sequences (REPs), which are at the same time highly overrepresented in host genomes. We have shown that this type of organization (REP-*rayt*-REP, Figure 1B) has been preserved during evolution and that both RAYTs and REPs undergo long-term coevolution. Characteristic structural elements in both RAYT and REP sequences suggest that all detected RAYTs and REPs are descendants of a common ancestor. We propose that their origin dates to the period after branching of the gammaproteobacteria, since no homologs have been found in other major bacterial lineages.

The structure of a *rayt *gene flanked by two oppositely orientated REPs is strikingly reminiscent of the organization of a typical bacterial insertion sequence. The position of REPs as terminal sequences for RAYT-encoding genes is supported by the fact that they are in many cases located very close or even immediately downstream of the *rayt *gene stop codon (Additional file 4), excluding additional terminal sequences. There are other known transposase genes associated with REPs, however, all of them are contained in *bona fide *ISs, complete with their own terminal sequences [27-34]. These ISs use REPs as targets for their transposition.

We have not found typical signs of IS-like mobility for RAYTs, *i.e. *presence of their multiple copies in host genomes and changes of chromosomal location. This might indicate that RAYTs have lost the ability to transpose their own genes. Still, there are at least two reasons to assume that RAYTs recognize REPs and cleave DNA strand in their proximity. By mere analogy, transposases always bind and cleave sequences that flank their genes during the course of transposition. This precise positioning of REPs by *rayt *genes is conserved. Moreover, related IS*200*/IS*605 *transposases recognize stem-loop structures [4,5] that can readily arise from imperfect palindromes like those contained in REP sequences.

One of the most interesting outcomes of this study is the previously unrecognized wide distribution of BIME elements. BIMEs were detected in most of RAYT- and REP-carrying species (Figure 2 and data not shown). Apparently, there is a common mechanism of BIMEs formation. The mechanism is targeted to *rayt *genes, one third of which are directly associated with BIMEs (Table 1). Furthermore, 3´ termini of *rayt *genes were found captured between REPs in four *rayt*-adjacent BIMEs (Additional file 4). BIMEs are known to exhibit extensive interstrain differences in length and distribution [24,27] that seem unlikely to result solely from processes such as homologous recombination or DNA polymerase strand-slippage. We hypothesize that the putative RAYT-catalyzed reaction, as described further, may pose the driving force behind BIME establishment and dynamics.

**Figure 6:**
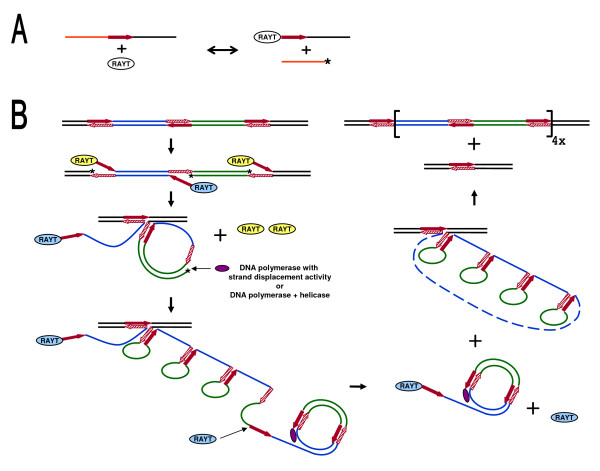
**Model of RAYT action**. (A) REP-specific DNA cleavage and ligation - scheme of hypothetical reaction (B) Model of RAYT-dependent BIME proliferation. REPs are represented with red arrows at corresponding DNA strands. REP-complementary sequences at opposite strands are represented with striped arrows. Two inter-REP segments are denoted in green and blue, respectively. RAYTs that form one dimer are denoted in the same color. Asterisks denote free OH groups. For details about the model, see text.

In the simplest case, the information contained in a REP sequence would be sufficient for its recognition and cleavage by RAYT. Because of high level of conservation of the 5´ head sequences (Table 1), we hypothesize that they might serve as determinants of position of cleavage site. Presumed REP-targeted RAYT activity would then result, for example, in reversible formation of a free hydroxyl group and covalent attachment of 5´ terminus of REP sequence to RAYT protein (Figure 6A). Host genomes typically harbor hundreds of REPs and all of them present potential substrates for RAYTs. The RAYT activity can thus account for various imaginable DNA rearrangements. There are two important aspects of presumed RAYT catalysis. Firstly, the transiently present free hydroxyl group can serve as a primer for initiation of DNA replication. Secondly, *in trans *ligation (reverse RAYT-catalyzed reaction) might occur relatively frequently between two RAYTs that act on different REPs. Because the assembly of catalytic site in related IS*200*/IS*605 *transposases is achieved by dimerization (due to their limited size), RAYTs probably form dimers as well. The physical proximity of two subunits enhances the frequency of *in trans *ligations.

We suggest that REP-dependent RAYT activity is responsible for some of the unusual observations regarding REPs. For example, high number of REPs in host genome is conditioned by presence of their cognate RAYT (Table 2). Further, the substitution rate for *rayt *genes was shown to greatly exceed the rate of substitutions in surrounding host genes (Figure 5). If RAYTs cleave in adjacency of their flanking REPs, resulting OH groups may prime DNA replication into *rayt *gene, leading to partial or complete replacement of one or both strands. When several rounds of such replication are performed during each cell cycle, excessive mutations accumulate. Although this is a rather complicated theory, the alternatives, like strong positive selection for mutated RAYTs, are equally uneasy to substantiate.

Another process we propose is RAYT-dependent is the preferential formation of new REPs in vicinity of a *rayt *gene (Additional file 3), following its horizontal transfer into the host. In this case, acquired RAYT obviously causes new REPs´ production, possibly through multiplication of existing REPs flanking its gene.

A possible model of BIME formation is depicted in Figure 6B. Starting with one basic module of BIMEs (two directly repeated REPs and one REP between them in inverted orientation - Figure 2), RAYT dimer cleaves at both top-strand REPs. Another RAYT dimer works on bottom strand, due to presence of single REP, only one unit of the dimer is attached to REP after cleavage. Upon *in trans *ligation within the frame of "yellow" dimer, circularized basic module and bottom strand hold together by their complementary parts. The circle is primed by the free OH group resulting from RAYT cleavage of bottom strand. At this point, rolling circle replication of basic module begins. The main replicative DNA polymerase (Pol III holoenzyme in *E. coli*) might accomplish the process on its own, since it was shown to possess intrinsic moderate strand-displacement activity [35]. The amplified basic module (BIME) is cut off from the rolling circle after the second unit of "blue" RAYT dimer cleaves newly synthetized REP. Then, second *in trans *ligation within the frame of "blue" dimer integrates BIME into the bottom strand. Following replication of chromosome and separation of daughter cells, one of them contains a modular BIME in its genome.

Taken together, we have gathered considerable amount of *in silico *evidence to propose significant role of transposases in generation of bacterial intergenic repeats. If our assumptions are true, then REPs and BIMEs represent a novel class of nonautonomous TEs. To confirm this, additional experiments are needed to simulate interaction between RAYTs and REPs *in vivo *and *in vitro*.

## Conclusions

Our findings offer an alternative approach for rapid identification of REPs in gammaproteobacterial genome sequences. Putative RAYT homologs can easily be found by a simple BLAST of conserved C-terminal part of any of known RAYTs against particular genome sequence. Invertedly positioned REPs can then be located flanking the *rayt *gene. Known REPs proved to be a useful tool for typing of intraspecific isolates, with high discriminatory power due to extensive REP dynamics [16,36]. REP typing is, compared to other methods, very fast and inexpensive, since it only requires one PCR, run from REP-complementary primers against chromosomal DNA template.

Upon determination of REP sequences, BIMEs can readily be identified in host genomes. Since BIMEs exhibit exceptional length polymorphism, they have been utilized as reliable markers for strain determination. As in previous case, the procedure is advantageous because of its quickness and simplicity [24,27].

## Methods

Bacterial genome sequences were downloaded from NCBI web site [37].

Direct and inverted repeats in *rayt*-flanking sequences were looked up with OligoRep [38].

REPs position and total number determinations and graphical plots were performed using pDRAW32 [39].

Multiple protein sequence alignments were constructed using MCOFFEE [40]. Phylogenetic trees were constructed using Drawtree or Drawgram applications from MOBYLE package [41]. Protein sequence trees were constructed from template CLUSTALX tree files (.ph) generated during MCOFFEE alignment. REP sequence trees were constructed from CLUSTALX guide tree files (.dnd) after being aligned with CLUSTALW [41].

## Abbreviations

BIME: Bacterial Insterspersed Mosaic Element; IS: Insertion Sequence; MITE: Miniature Inverted repeat Transposable Element; RAYT: REP-Associated tYrosine Transposase; RC: Rolling Circle; REP: Repetitive Extragenic Palindrome; TE: Transposable Element

## Authors' contributions

JN carried out the analyses and wrote the manuscript. TH identified horizontal transfer in tetracycline-resistant strains. IL supervised the work and critically read the manuscript. All authors read and approved of the final manuscript.

## Supplementary Material

Additional File 1 **Distribution of REP sequences in genomes of selected bacteria.** REP coordinates, orientation and number of mismatches with respect to REP sequences in Table 1 are indicated. If dimorphic REPs appertain to a given RAYT (for example in *Enterobacter sakazakii*), they are denoted as REPA (upper line in Table 1) and REPB (lower line in Table 1). Click here for file

Additional File 2 **Phylogram of all RAYT proteins listed in Table**1**with reference IS*200*/IS*605 *transposases as outgroup**. Putative root of phylogram is denoted with a star. Click here for file

Additional File 3 Examples of colocalization of REP sequences with *rayt *genes. Click here for file

Additional File 4 **Incorporation of *rayt *gene 3´ terminus into BIME.** REPs are highlighted in red, their GT(A/G)G head is in bold and blue. *rayt *gene is denoted in italics, bold and underlined.
Inter-REP segments are highlighted in blue and yellow, or gray, or green, respectively. Click here for file
