# Supplementary material for: Identification and characterization of repetitive extragenic palindromes (REP)-associated tyrosine transposases: implications for REP evolution and dynamics in bacterial genomes
Source: BMC Genomics. 2010 Jan 19;11:44. doi: 10.1186/1471-2164-11-44 (PMC2817692; doi:10.1186/1471-2164-11-44)
Supplement: Additional File 1 — Distribution of REP sequences in genomes of selected bacteria. REP coordinates, orientation and number of mismatches with respect to REP sequences in Table 1 are indicated. If dimorphic REPs appertain to a given RAYT (for example in Enterobacter sakazakii), they are denoted as REPA (upper line in Table 1) and REPB (lower line in Table 1). [file 1471-2164-11-44-S1.PDF]

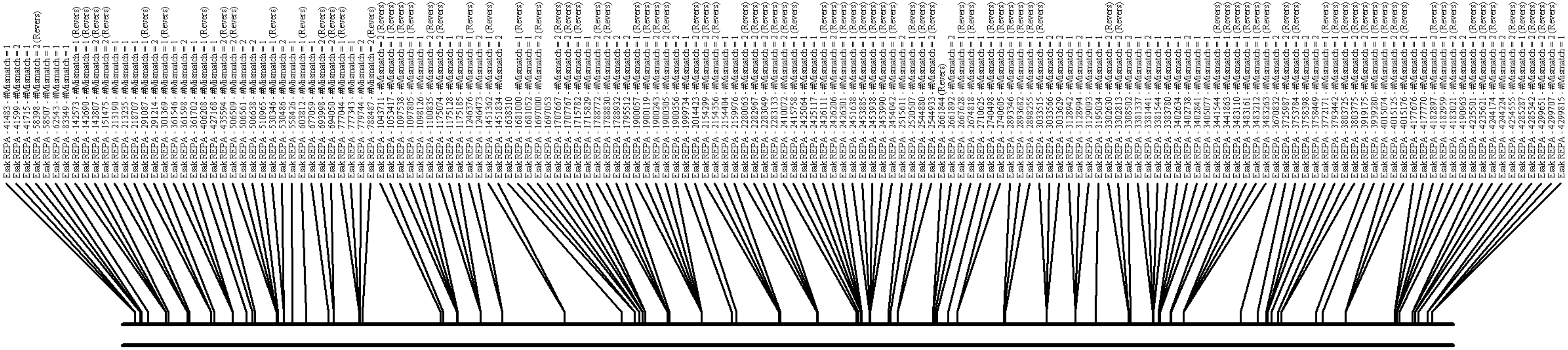

Enterobacter sakazakii ATCC BAA-894
4368404 bp

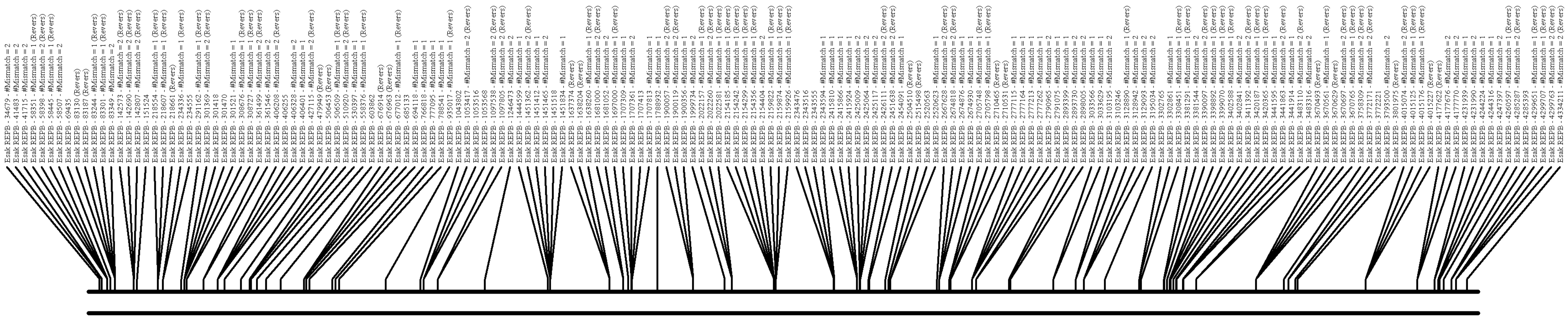

Enterobacter sakazakii ATCC BAA-894
4368404 bp

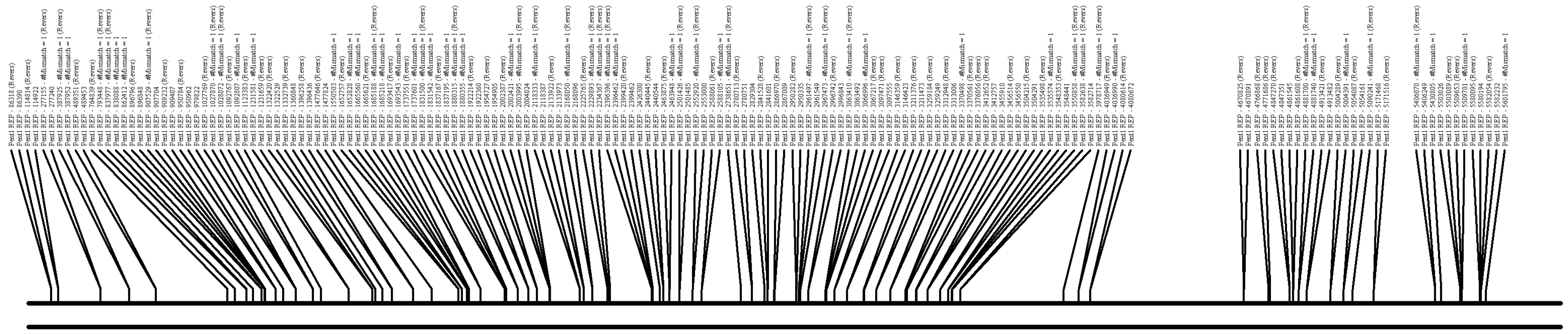

Pseudomonas entomophila L48
5888801 bp

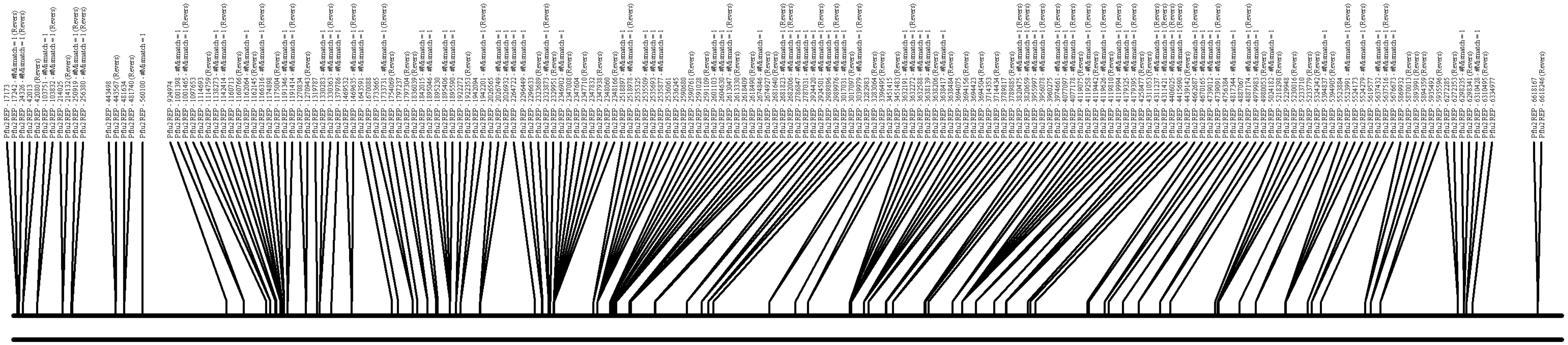

Pseudomonas fluorescens SBW25
6722563 bp

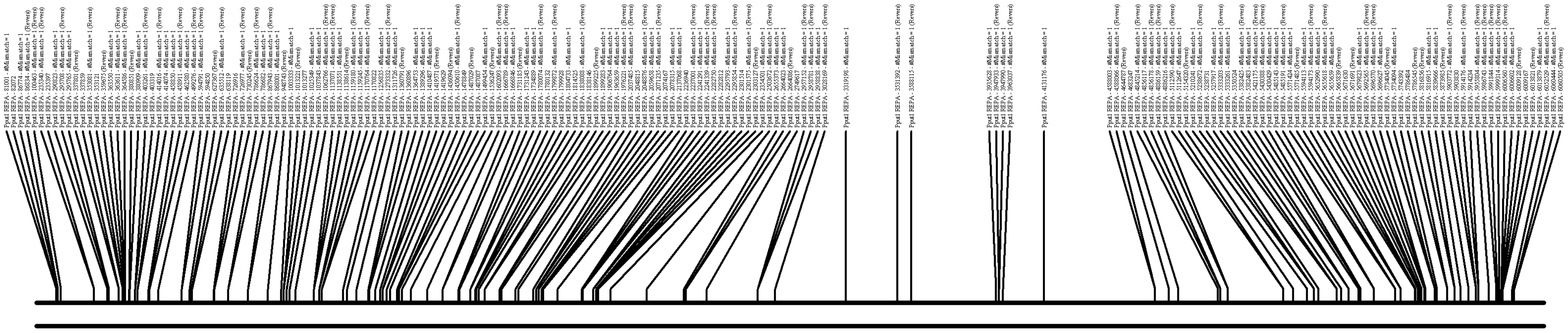

Pseudomonas putida KT2440
6181884 bp

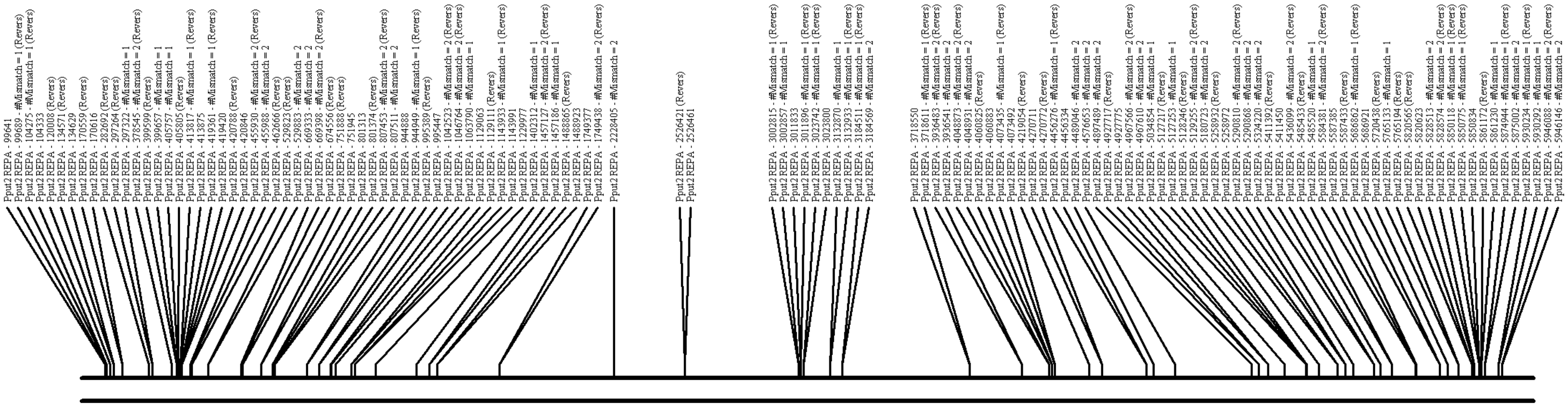

Pseudomonas putida GB-1
6078451 bp

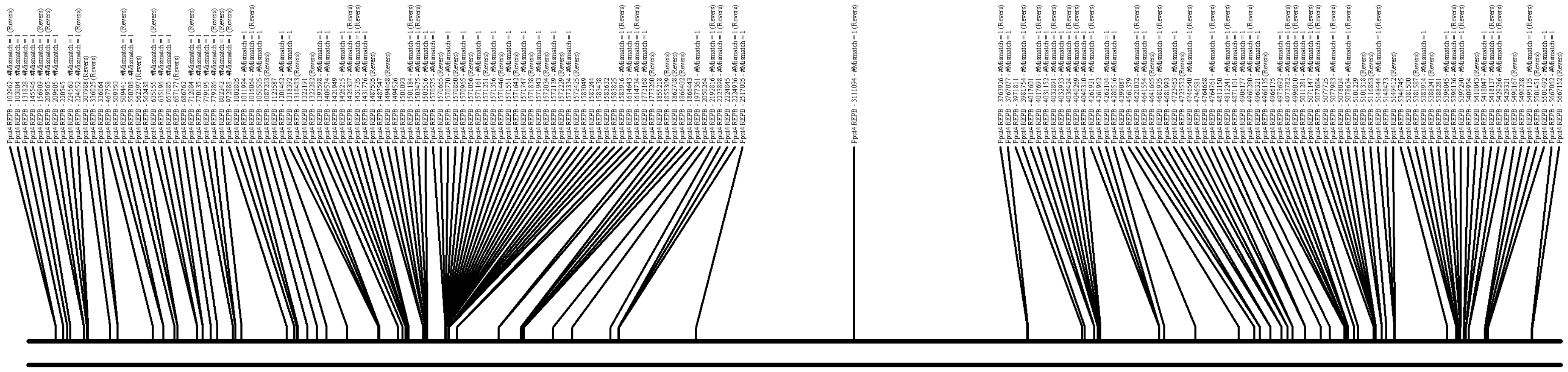

Pseudomonas putida W619
5774350 bp

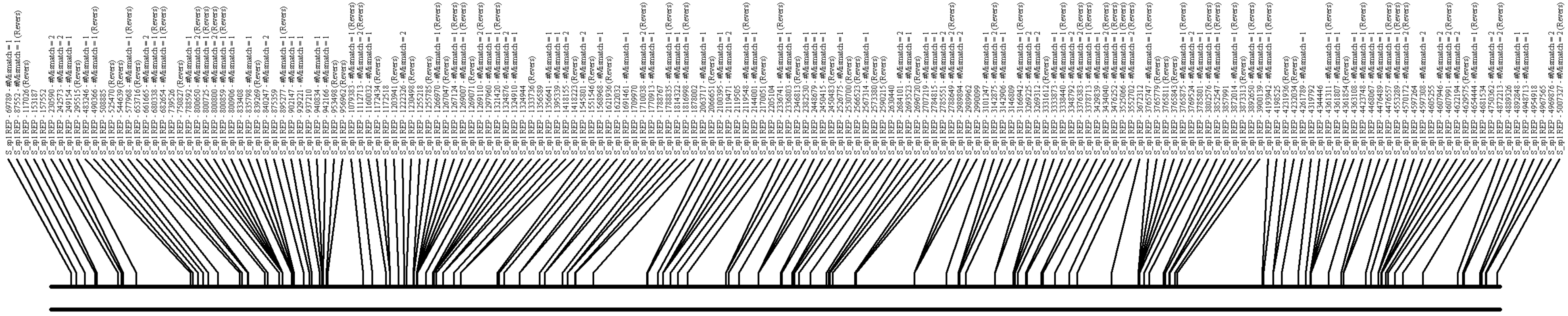

Stenotrophomonas sp. SKA14
5017786 bp

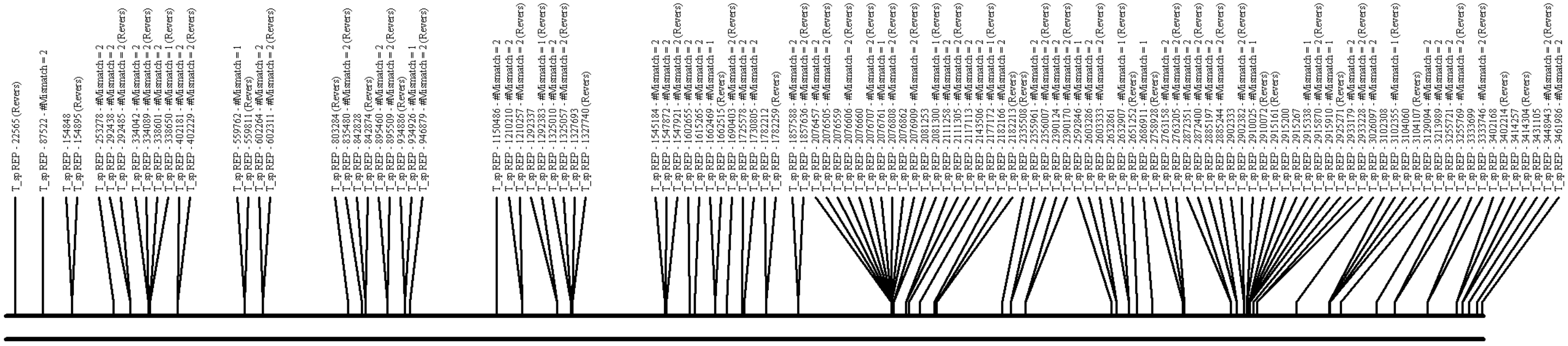

Thioalkalivibrio sp. HL-EbGR7
3464577 bp

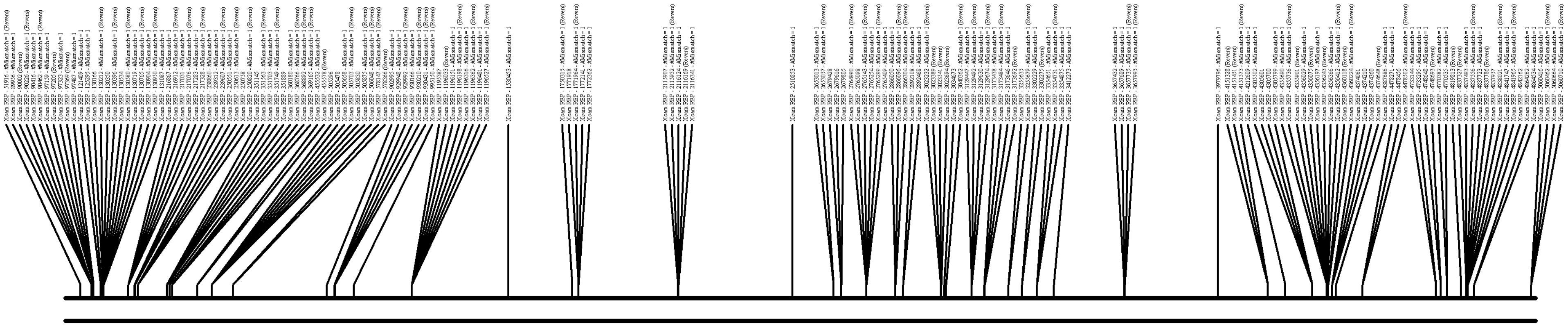

Xanthomonas campestris pv. campestris str. ATCC 33913
5076229 bp
