# Supplementary figures and images for: Identification and characterization of repetitive extragenic palindromes (REP)-associated tyrosine transposases: implications for REP evolution and dynamics in bacterial genomes
Source: BMC Genomics. 2010 Jan 19;11:44. doi: 10.1186/1471-2164-11-44 (PMC2817692; doi:10.1186/1471-2164-11-44)

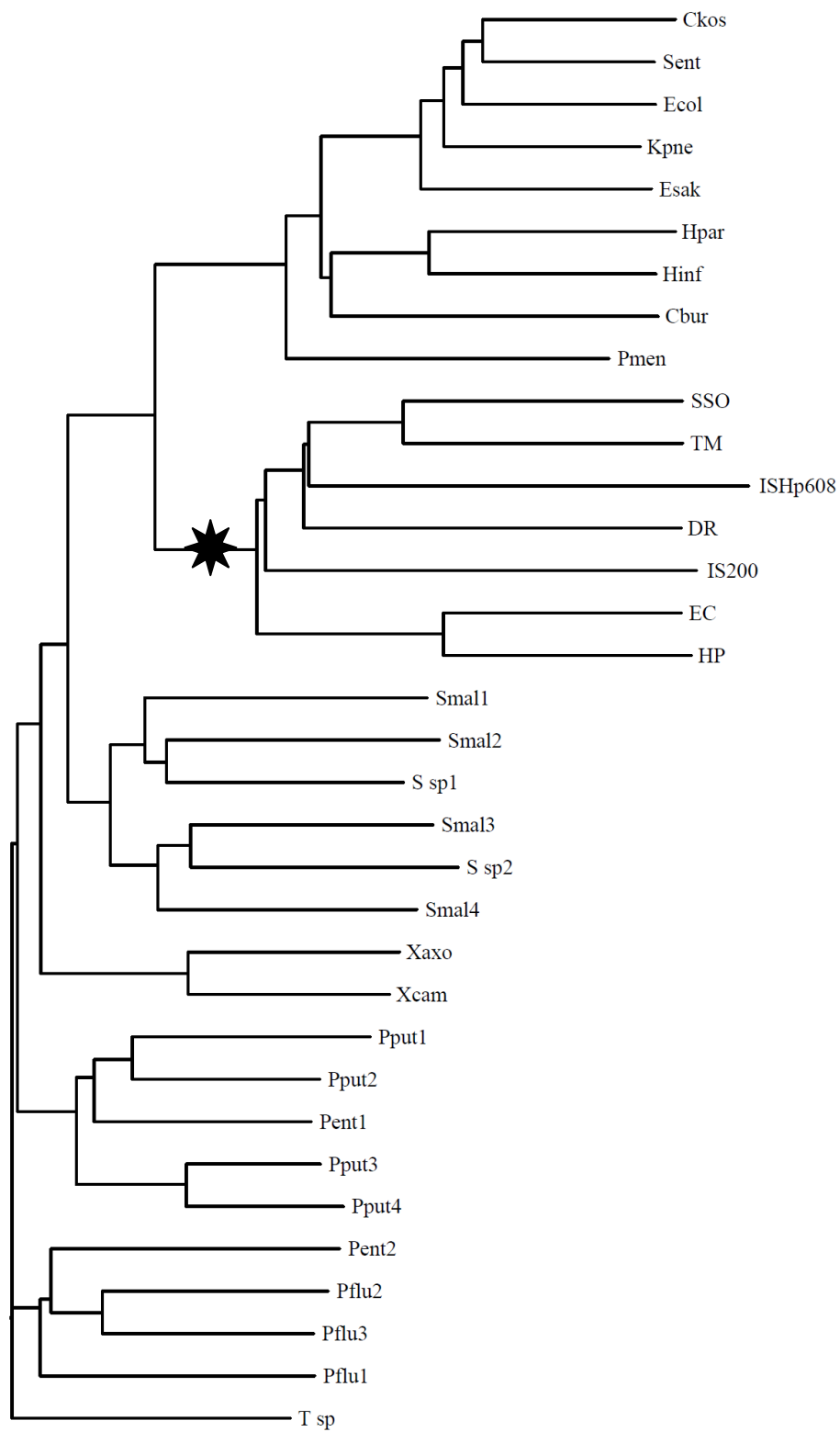

Supplement: Additional File 2 — Phylogram of all RAYT proteins listed in Table1with reference IS200/IS605 transposases as outgroup. Putative root of phylogram is denoted with a star. [file 1471-2164-11-44-S2.PDF]
