# Supplementary material for: Identification and characterization of repetitive extragenic palindromes (REP)-associated tyrosine transposases: implications for REP evolution and dynamics in bacterial genomes
Source: BMC Genomics. 2010 Jan 19;11:44. doi: 10.1186/1471-2164-11-44 (PMC2817692; doi:10.1186/1471-2164-11-44)
Supplement: Additional File 3 — Examples of colocalization of REP sequences with rayt genes. [file 1471-2164-11-44-S3.PDF]

Host strain	REP sequence	*rat* coordinates
Idiomarina loihiensis L2TR	T**GTAG**CCTGACATTTATGTCAGG	c533882-533451
Marinomonas sp. MWYL1	T**GTAG**GTCGGCCTTTAGGCCGTCAA	c865868-865416
Shewanella woodyi ATCC 51908	**GTAG**GTCGGCATTTATGCCG	c2093726-2093277
Thauera sp. MZ1T	**GTGG**GAGCGACGCAAGTCGCGAT	c1245166-1244705

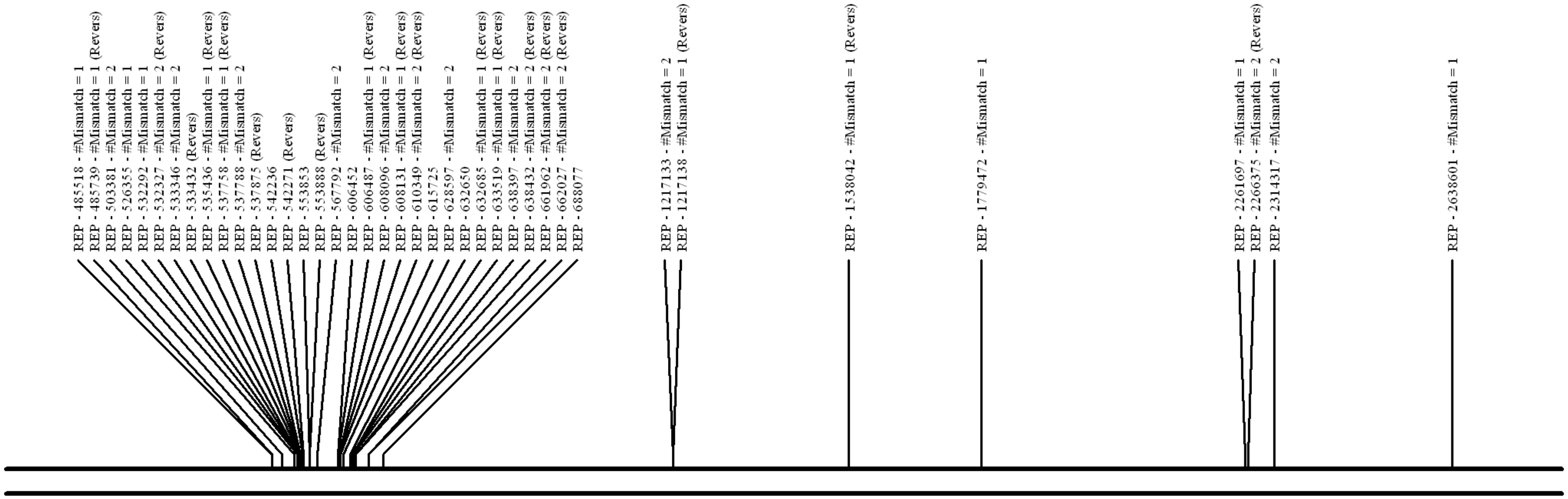

Idiomarina loihiensis L2TR
2839340 bp

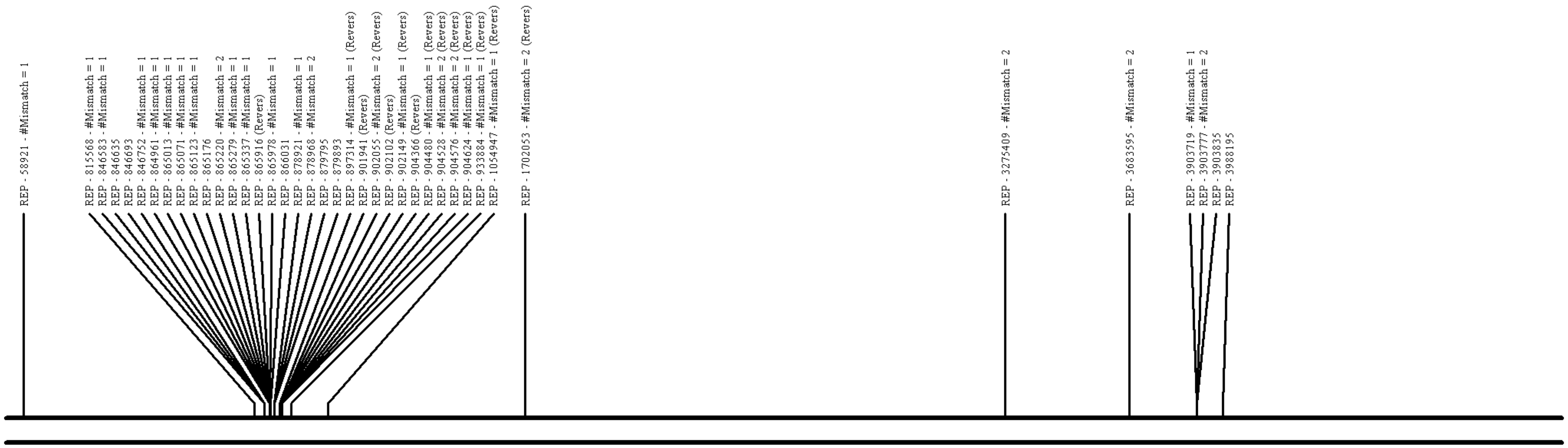

Marinomonas sp. MWYL1
5100366 bp

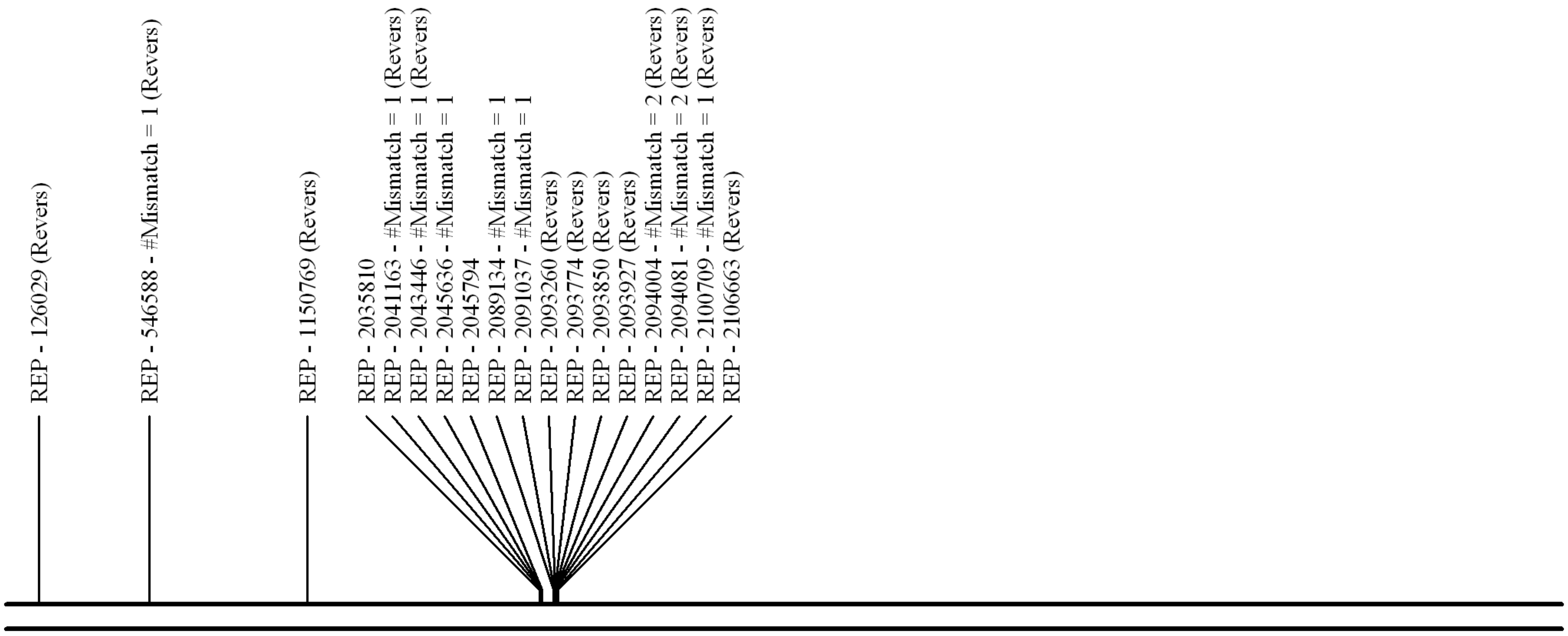

Shewanella woodyi ATCC 51908
5935426 bp

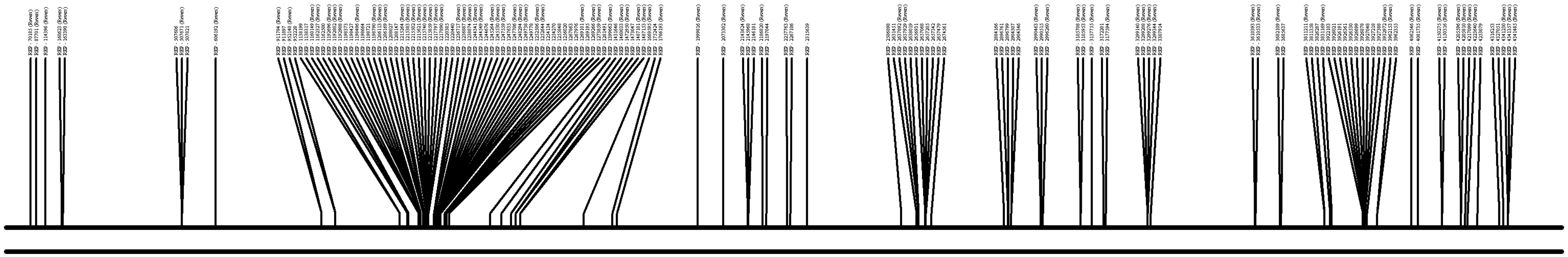

Thauera sp. MZ1T
4496230 bp
