# Supplementary material for: Identification and characterization of repetitive extragenic palindromes (REP)-associated tyrosine transposases: implications for REP evolution and dynamics in bacterial genomes
Source: BMC Genomics. 2010 Jan 19;11:44. doi: 10.1186/1471-2164-11-44 (PMC2817692; doi:10.1186/1471-2164-11-44)
Supplement: Additional File 4 — Incorporation of rayt gene 3´ terminus into BIME. REPs are highlighted in red, their GT(A/G)G head is in bold and blue. rayt gene is denoted in italics, bold and underlined. Inter-REP segments are highlighted in blue and yellow, or gray, or green, respectively. [file 1471-2164-11-44-S4.PDF]

Pput4

>gi|170719187:c5078711-5077456 ***Pseudomonas putida W619***, complete genome
GATGCATTGCGCCTGGGGCTGACCCGGTCTTCCTACCTGTCGTCGTGATTGTCACGGCCGCTCCTGCTAC
CCCTGTAGGAGCGGCCTTGCGTCGCGAATGAGGGCGCAGCCCTCACCGGTGTCACATCAAGAAAAGGAAC
AAACGTACCCGGCTGCATGGGGCTGACTACCGTTGAACGCAATGTTCAAGGGATACCGAC***ATGGACCGTC***
AAGGCAATCACCGTTTGCGCAAAGGCCGCCACTCGGAGCAGGGGAGGCTGTATCTATTAACAACGGTTAC
CCGCAATCGCGCTCCAATCTTCAAGAATCTTCCTTTCGCCCGAGCCGCCATTCAACAGTTGCGGCTAAGC
GATCAGGAAGCGTGCTGTCGAACGCTAGCGTGGGTCTTGATGCCAGATCATCTGCACTGGCTGATCGAGC
TAGGCCACGGAACGCTTGGCGAATTGATGTGCGCATTCAAATCTCGGAGCAGTTGCGCGCTTTATCGAGC
CGGAGCTGATAGGCGTCGTATCTGGCAACCTGGCTTCCATGACCGAGCGTTACGGCGTGAGCAGGACATC
AGGGCCGTTGCTCGATACATCATAGCCAACCCAATCCGTGCCGGACTGGTACAGCGTGCTGGCGAGTATT
CTCACTGGGATTGTGTGTGGCTGTGAGGCTGCAGATGAGGGCTGCGCCCTCATTTCGCGACACAAGGCCG
CTCCTACAGCGGTACCGCGTCGCCTGTAGGAGCGGCCTTGCGTCGCGAAAGGGCTGCAAGGCAGCCCCAG
CATCAGTGAATCCACAGTCTTTTTCGTTGCAGGCGCCAACCCACCCATCCCAACGTCAGCGCCCGCAACG
CCATGAACCCGAGAAACGCAAACCACAAGCCGTGGTTGCCAAACCCACTC***CAATCCGTGCCGGACTGGTA***
CAGCGTGCTGGCGAGTATTCTCACTGGGATTGTGTGTGGCTGTGAGTATGCAGATGAGGGCTGCGCCCTC
ATTTCGCGACACAAGGCCGCTCCTACAGCGGTACCGCGTCGCCTGTAGGAGCGGCCTTGCGTCGCGAAAG
GGCTGCAAGGCAGCCCCAGCATTAGTGAATCCACAGTCCTTTTCGTTGCAGGCGCCAACCCACCCATCCC
AACGTCAGCGCCCGCAACGCCATGAACCCGAGAAACGCAAACCACAAGCCGTGGTTGCCAAACCCACTCA
TGGCCCACGCCAGCGGCAATGCAACCAGCACCGAAACCAGCATTGCATTGCGCATCTCCCGCGCCC

Pflu2

>gi|229587578:2346865-2348266 ***Pseudomonas fluorescens SBW25***, complete genome
TGAGGTGTTTTCTTTGGCGAGGGAGCTTGCTCCCGCTGGGCTGCGAAGCGGCCCCAGTTCATACGCCACG
TTCGTTCAGGTCATTCAGGGTTTAGCTTTTTGGGGCTGCTGCGCAACCCAGCGGGAGCAAGCTCCCTCGC
CACAAGGGGTGGAAGGAAATATGAGGAAATTGAATTGAGCAGGGAGATGGGCGCGGACTGCTAGGTTGAG
TTGATCAGATTATCAATCGCCAGGAGCATTGAGTTGCTTCAGAAGCCCAGCTCTCATTTGTTACGTCGTG
GTCGTTACTCAGAAACAGGTCGTGCCTACTTGGTGACCGCGGTGGTTTATCAACGGCGGCCCGTATTTAC
CCACTTTTACCTGGGACGGTTACTGGTTGCAGAACTCAAGCAGGCCCATGACCTCGGCCTGGTTGAATCA
CTCGCCTGGGTCATCATGCCGGATCATTTCCATTGGTTGTTTGAATTGCAGGACGCGTCGCTGCCCCAGG
TTATGCAGCGGGTGAAATCGAGGAGTACGCTGACGATCAATCGAGCCTGCGGCACACAGGGCGCTTTTTG
GCAAAGCGGATACCATGATCGAGCCGCGCGTGCCGAAGAAGACCTTATCCAAATCGCGCGCTACATCATC
GGTAATCCCCTGCGAGCAGGCTTGGTGGATCAAGTGGGTGACTACCCTCTATGGGACGCTACCTGGCTAT
GAAAGCCTGTCACCTGTGGCGAGGGAGCTTGCTCCCGCTGGGCTGCGAAGCGGCCCCAATTCATACGCCG
CGTTCGTTCAGGTCATTCAGGGTTTAGCTTTTTGGGGCTGCTGCGCAACCCAGCGGGAGCAAGCTCCCTC
GCCACGGGGGTGGTGCGCTACATTGTCGGTAATCCCGTGCGAGCAGGCTTG***GTGGGTGACTACCCTCTAT***
GGGACGCTACCTGGTTATGAAGGCATGTCACCTGTGGCGAGGGAGCTTGCTCCCGCTGGGCTGCGAAGCG
GCCCCAACTCCTACGCCGCGTCTGTTCAGGGAGTACACGGTTTTGCTTTTTGGGGCTGCTGCGCAACCCA
GCGGGAGCAAGCTCCCTCGCCACGGGGGTGGTGCGCTACATTGTCGGTAATCCCCTGCGAGCAGGCTTG***G***
TGGGTGACTACCCTCTATGAGGCGCTGCCTGGCTATGAAGGCATGTCACCTGTGGCGAGGGAGCTTGCTC
CCGCTGGGCTGCGAAGCGGCCCCGACTCTTACGCCGCGTCTGTTCAGGGAGTACACGGTTTTGCTTTTTG
GGGCTGCTGCGCAACCCAGCGGGAGCAAGCTCCCTCGCCACAGGTGGCGTGTTGCTTGATTGAATGATCG
TGCGTGGCAGCGGTCCGAACCTGGGTACGCATCATTCATTGAAGGAGCACCCATGAAATCCAAATCCCTG
AT

Smal4

>gi|190572091:c2850131-2847800 ***Stenotrophomonas maltophilia K279a***, complete genome

CACCCTGCGCGGCTGAGCCCCGACGGCCGCCTGCCGGCAGTGGTAGTGCCGGCCGCTGGCCGGCAATCCA
TAGTCCCGGTAGTGCCGGCCGCTGGCCGGCAACCCACAGCTCCGGTAGTGCCGGCCGCTGGCCGGCAACC
TCATTCAACTTCAGACATCTCCCAACCTCCATCGAGGCATGGCCCGACAGCCAACGCCCA***ATGATCGACC***
CACCATGGCGCATGAGCAGCCATCGCCTCCGTCTGGGCCGTCACTCGATCATCGGCCAGTCCTACGTTCT
GACAACCACGACACACCAGCGCCGCCGGCTCTTTGAAAGCGAAGCTGCAGCAGCATGTGTAATCGACCAG
TTCCACTACATCGAACAGCGCGGGCTCGTACAGTCACACGCATGGGTCGTCATGCCGGATCACGTCCACT
GGATGTTCGAGCTGCGCGCAGCTCACCTTCCCGACATCGCGCGCCGGATGAAATCGTCGAGCGCGCTCGC
CCTGAATCGCCTGGTGGGGCGTCGATGTACGGTCTGGCAGTCTGGCTACTTTGATCATGCCGTGAGGGCC
GAGGAGTCGCTGGCGCAGCAAGCGCTGTACATCCTGGGGAATCCGGTGCGCGCCGGTCTTGCTGGGCAGA
TTGGCGAGTATCCGTATGCATGGTCGGTCTGGTTGTGATGCCGGCCAGCGGCCGACACTACCAAAGCAAG
GGCGAAGCGCAGCGCAGATTGCGTTCCGGCACACAGAGCAGCCTCGATGGTAGTGCCGGCCGCTGGCCGG
CAGCCATGCATCTCCGCGCGGGTCATGGGGTTGCCGGCCAGCGGCCGGCACTACCAGTGAATGTTCAGCG
CAGCCATTCACTGTTCAGCGCCGCTTGCGCGCGCCTCGGCCG***TGGTCGGTCCGGTTTGA***TGCCGGCCAGC
GGCCGGCACTACCACAGCAAAGCCGAAGCGCAGCGCAGATTGCGTTCCGGCGCACAGAGCACCCTCGGTG
GTAGTGCCGGCCGCTGGCCGGCAACCCCATGACCCGCGCGAAGATGCATCGCTGCCGGCCGGCAGCTGGC
ACTACCAGTCGATGTTCAGCGCGGCCATTCGCTGTTCAGCGCCGCTTGCGCGCGCCTCGGCCG***TGGCCGG***
TCTGGTTGTGATGCCGGCCAGCGGCCGGCACTACCAAAGCAAATGCGAAGCGCAGCGCAGATTGCGTTCC
GGCACACAGAGCAGCCTCGATGGTAGTGCCGGCCGCTGGCCGGCAACCCCATGACCCGCGCGGAGATGCA
TGGCTGCCGGCCGGCAGTCGGCACTACCAGTCGATGTTCAGCACGGCCATTCGCTGTTCAGCGCCGCTTG
CGCGCGCCTCGGCCG***TGGTCGGTCTGGTTGTGA***TGCCGGCCAGCGGCCGACACTACCAAAGCAAGGGCGA
AGCGCAGCGCAGATTGCGTTCCGGCACACAGAGCAGCCTCGATGGTAGTGCCGGCCGCTGGCCGGCAACC
CCATGACCCGCGCGGAGATGCATGGCTGCCGGCCAGCGGCCGGCACTACCAGTGAATGTTCAGCGCAGCC
ATTCACTGTTCAGCGCCGCTTGCGCGCGCCTCGGCCG***TGGCCGGTCTGGTTGTGA***TGCCGGCCAGCGGCC
GGCACTACCAAAGCAAATGCGAAGCGCAGCGCAGATTGCGTTCCGGCACACAGAGCAGCCTCGATGGTAG
TGCCGGCCGCTGGCCGGCAACCCCATGACCCGCGCGGAGATGCATGGCTGCCGGCCGGCAGTCGGCACTA
CCAGTCGATGTTCAGCACGGCCATTCGCTGTTCAGCGCCGCTTGCGCGCGCCTCGGCCG***TGGTCGGTCTG***
GTTGTGATGCCGGCCAGCGGCCGGCACTACCACAGCAAAGCCGAAGCGCAGCGCAGATTGCGTTCCGGCG
CACAGAGCGCCCTCGGTGGTAGTGCCGGCCGCTGGCCGGCAACCCCATGACCCACGCGGAGATGCATAGC
TGCCGGCCAGCGGCCGGCACTACCAGTCGATGTTCAGCACGGCCATTCGCTGTTCAGCGCCGCTTGCGCG
CGCCTCGGCGA***TGGTCGGTCTGGTTGTGA***TGCCGGCCACCGGCCGGCACTACCACAGCAAAGCCGAAGCG
CAGCGCAGATCGCGTTCCGGCGCGCAGAGCACCCTCGGTGGTAGTGCCGGCCGCTGGCCGGCAACCTCAT
GACCTGCGCGGAGTTGCATGGCTGCCGGCCAGCGGCCGGCACTACCAATTGGTGTTCAGCGCGGCTTGCG
TGTCCGCTTCGGCTTCGCGGTATCTGCACCGCTGGCGGCGCCGTCCTGCAGCGCCTGCAGCCACGACAAC
GTATCCACATAGTCGATGAAGT

>gi|52424055:626446-628194 ***Mannheimia succiniciproducens MBEL55E***, complete genome
TGGCGTAATTTAGACAGCCAAATCAAAGAAAATATGGAAAAACATACCGCACTTTCAGTTCTCGCCGAAC
AGCCGACATTAATCAAACGCCCTATTATCCTGCAAGACGGCATCGCATTAATTGGTTTTAATATAAAAGA
ATATAAAAAAAGCTTTTGGTTAATTACGTAGGGTGGTCTTGCCGGCCCACCAATAATAAGCATGG***ATGATA***
TTATGTCAAATTATCGAAGAGATTTTTCACCGGGTGCAACTTATTTTTTTACCGTTGTTATAAACCAACG
TAGTGACGGGCTTCTAATAAAATATATAAATGAGTTTAAGCAAGCTTATCAAGATGTTGTTTCATATTAT
CCATTTGAAACAATTGCACTAACAGTATTACCTGATCATTTTCATTTAATTATGCAATTACCAGAAAATG
ATTCAGATTATTCAAAAAGAATTTCCTCTTTAAAATATAATTTTAGTTCATTATTACCAACTTATTATCG
TAATATGAATTTGAGTCGTCAGTTCAAACGGGAAGCAGGTATTTGGCAACGGCGATTTTGGGAACATTTG
ATCAGAGATGACAGAGATTTGGATAATCATATTGATTACGTGTATTACAATCCTGTAAAACATGGTTATG
TATCTCAAGTTATGGATTGGAAATATTCAACTTTTCATCGAGATGTTAAAAATGGGATTTTTGAATTAGA
TTGGGGAAGTTATATTTCTGAATCTGTCCGGAATTTATATTTGGATTAATTATGAAATTGGTGGGCTGGC
AAGCCCACCCTACGCGTAAGAATAAAAAACAAACGAAAAAACACAATGATTAATTTTGCACGGTATCCTA
TTAACTGGTTTTTGATATAAAAGAATATAAAAAATTTTGGTTAATCGTATATCGTGTAGGGTGGGCTTGC
CAGCCCACCCTACGCGTAAGAATAAAAAACAAACGAAAAAACACAATGATTAATTTTGCACGGTATTCTA
TTAACTGGTTTTGATATAAAAGAATATAAAAAACTTTTGGTTAATTGTATATCCGTGTAGGGTGGGCTTG
CCAGCCCACCGGCAAGCTT***TCGAGATGTTAGACATAGGATTTTTGAATTAGATTGGGGAAGTTGTATTTC***
TGAATCTGCCCGGAATTTATATTTGGATTAATTATGAAATTGGTGGGCTGGCAAGCCCACCCTACGCGTA
AGAATAAAAAACAAACGAAAAAACACAATGATTAATTTTGCACGGTATCCTATTAACTGGTTTTGATATA
AAAGAATATAAAAACGCTTTCGGTTAATTGTATATCCGTATAGGGTGGGCTTGCCAGCCCACCGGTAAGC
TT***TCGAGATGTTAAACATAGGATTTTTGAATTAGATTGGGAAAGTTATATTTCTGAATCTGCCCGGAAAT***
TTTATTTGGATTAATTATGAAATTGGTGGGCTGGCAAGCAGACCCTACGCGTAAGAATAAAAAACAAACG
AAAAAACACAATGATTAATTTTGCACGGTATCCTATTAACTGGTTTTGATATAAAAGAATATAAAAAAAC
TTTTGGTTAATCGTATATCCGTGTAGGGTGGGCTTGCCAGCCCACCGGCTACGAGTGAGAATAAAAAATA
AATGAAAAACACAATTATCAATCTTGCACAGGATTTAATCCGTCGTCCTTCAATTAGCCCGGATGATCAA
GGGTGCCAGCAGGTTATTGCAGAGCGTTTAACAAAGCTGGGCTTTAACATTGAATGGATGTCTTTTAAC
